# Force variability of thoracic spine mobilization and manipulation delivered by experienced physiotherapists to healthy human volunteers and a manikin: an observational study

**DOI:** 10.1186/s12998-025-00619-7

**Published:** 2025-12-09

**Authors:** Nathalie Thurnherr, Petra Schweinhardt, Lindsay M. Gorrell

**Affiliations:** 1 Institute of Physiotherapy, School of Health Sciences, ZHAW Zurich University of Applied Sciences, Winterthur, Switzerland; 2 Integrative Spinal Research Group, Department of Chiropractic Medicine, Balgrist University Hospital, University of Zurich, Zurich, Switzerland

**Keywords:** Mobilization, Manipulation, Thoracic spine, Manikin, Biomechanics

## Abstract

**Background:**

Many health care professionals use spinal mobilization (MOB) and manipulation (MAN) to treat musculoskeletal disorders. Research shows advantages of learning these techniques using a manikin. However, the force–time characteristics of MOB and MAN applied to manikins may differ from those delivered clinically to humans. This study reports on differences between the force–time characteristics of MOB and MAN delivered by experienced physiotherapists to the thoracic spine of both humans and a manikin.

**Methods:**

Data were collected September–October 2023. Experienced physiotherapists applied prone MOB (Grade 3 central posterior-to-anterior, 30 s) and a single prone MAN to the T6 vertebra of three healthy human volunteers and a manikin with each volunteer-manikin pair representing one of three different patient scenarios (vignettes): vignette 1: 30-year-old male, 185 cm; vignette 2: 50-year-old male, 175 cm, and vignette 3: 65-year-old female, 165 cm. The applied forces were measured using a flexible pressure pad (100 Hz) and were compared descriptively between humans and the manikin.

**Results:**

Data were analyzed from 13 physiotherapists (seven females, age (median, IQR): 40 (36–45) years, experience as physiotherapist: 14 (12–21) years). Peak forces on the manikin were higher than on the humans. Specifically, for MOB, average mean peak force differences (95% confidence interval) were: vignette 1: 58N (36, 80); vignette 2: 99N (74, 124); and vignette 3: 50N (31, 68). Similarly, for MAN, average peak force differences were: vignette 1: 128N (79, 177); vignette 2: 147N (94, 199); and vignette 3: 137N (101, 172). For MAN, greater mean peak forces were applied on vignette 1 than vignette 3 on the human 355N vs 284N and on the manikin 483N vs 421N.

**Conclusion:**

In this study force–time characteristics of MOB and MAN performed by experienced physiotherapists on the thoracic spine of a manikin were different from those delivered to healthy humans: the forces applied to the manikin were higher for all vignettes for both techniques. However, forces were modulated to the vignette, both on the human and manikin.

**Supplementary Information:**

The online version contains supplementary material available at 10.1186/s12998-025-00619-7.

## Background

Musculoskeletal disorders, most commonly spinal pain, are the largest cause of disability globally [1, 2]. Such disorders cause considerable financial and societal cost to sufferers and society [3]. To treat these disorders, clinical practice guidelines recommend several evidence-based interventions [4–6] such as joint mobilization (MOB) and manipulation (MAN). These interventions have beneficial effects (e.g. hypoalgesia, increased range of motion) [7] when used to treat spinal pain disorders and are commonly used by health care professionals, including physiotherapists and chiropractors [4, 8, 9]. Although there seems to be no difference between the clinical benefits of MOB or MAN [4, 6, 10, 11], there are biomechanical differences. Specifically, MOB is defined as a manually applied oscillatory force with low velocity and variable amplitude, and is delivered with the intention of passively mobilizing the contacted articulation [12, 13]. Four main grades of MOB are described by Maitland and colleagues [12]: Grades 1 and 2 are movements which start at the beginning of range of motion before the first onset of resistance to movement (R1). Generally they are used for pain inhibition. Grades 3 and 4 are designated as movements to the point of limitation in range of movement (R2) and are commonly used to increase the range of motion. Three reviews [13–15] have reported on the force–time characteristics (e.g. peak force, duration of MOB, frequency, and force amplitude) of spinal MOB delivered in a posterior-anterior direction. All found a lack of systematic biomechanical quantification and large heterogeneity in the definitions of force delivery and force–time characteristics, making it difficult to build on previous research findings and to define appropriate dosage of biomechanical characteristics needed to treat a patient [16].

MAN is defined as a high velocity, low amplitude thrust technique delivered to the contacted articulation [17]. Spinal MAN can be quantified using characteristics such as total peak force, Downward Incisural Point (DIP), rate of force application and thrust duration [18–20].

In contrast to MOB, a guideline for the standardized reporting of these characteristics exists for MAN [21]. Nevertheless, considerable variability in quantifying these biomechanical characteristics has been reported [18–20]. Similar to MOB, it is not clear which dose of the biomechanical characteristics of a MAN are clinically relevant [18, 20, 22, 23]. However, biomechanical quantification of MOB and MAN delivered by experienced clinicians is essential to identify possible characteristics that could be related to clinical effectiveness in the future [14, 18, 22]. Furthermore, providing students with force–time characteristics delivered by experienced clinicians could also be important for the teaching of MOB and MAN, providing objective feedback and quantifying the intervention.

Additionally, research on the teaching and learning of spinal MOB and MAN identifies several advantages (e.g. objective feedback in combination with other mechanical aids, lower risk of negative side effects, safe to fail environment) of practicing manual techniques with a manikin or other mechanical training aid [22, 24–27]. However, no studies have been conducted to determine whether the MOB and MAN psychomotor skills trained on a manikin transfer into improved competency levels in a clinical setting with real patients. One study investigated the transferability of cervical MAN delivered to a manikin compared to a healthy human volunteer [28]. In this study, Duquette and colleagues reported that total peak forces applied to a human by chiropractic students were approximately 50% lower than those applied to a manikin. However, data were only reported for students and it is unknown if the force–time characteristics of experienced clinicians would differ from the study cohort.

The aim of this study was to investigate whether the force–time characteristics of MOB and MAN performed by experienced physiotherapists to the thoracic spine of a manikin were different from those delivered to healthy humans.

The authors hypothesized that clinicians would apply greater peak forces on the manikin than on human volunteers.

## Methodology

This study was an observational trial conducted at Balgrist University Hospital in Zurich, Switzerland. The study design was developed and reported in accordance with the STROBE guidelines for observational studies [29]. The study protocol was reviewed by the relevant ethics committee, and it was deemed that no ethical approval was necessary (BASEC-Nr. 2023-01094). Data were collected September–October 2023. All data were collected in accordance with the Declaration of Helsinki [30]. All participants (clinicians and volunteers) provided written informed consent prior to participating in the study and were asked to report adverse events that might be related to the interventions. An adverse event was defined as any new or worsening symptom (e.g., pain, discomfort, illness) occurring in a volunteer after a MOB or MAN intervention delivered during data collection and classified according Carnes et al. [31]. Volunteers themselves reported any such adverse events to the research team immediately and at a short-term follow-up.

### Participants: Clinicians

Experienced physiotherapists (> 5 years’ experience) who apply MAN to the spine at least once per week were recruited in private outpatient clinics, hospital physiotherapy departments and academic settings using convenience and snowball sampling (e.g. word of mouth, online advertising). Clinicians were excluded if they had any injuries or impairments that would affect the delivery of the treatment (e.g. pain in the hand, shoulder injuries) or if they were not able to understand written informed consent and instructions in English or German.

### Participants: Volunteers

Three healthy human volunteers were recruited to match with three fictional patient scenarios (vignettes). Each vignette consisted of a short patient history representing a case that is regularly treated in physiotherapy clinical practice (e.g. Additional Files 1–3) and that had previously responded well to manual therapy applied to the mid-thoracic spine. Vignette 1 was a young male (30 years, 185 cm), vignette 2 a middle-aged male (50 years, 175 cm) and vignette 3 an elderly female (65 years, 165 cm). Volunteers were normal bodyweight for height, asymptomatic, did not have pathology affecting the thoracic spine (e.g. bone diseases, cancer, recent surgery, or trauma) or any contraindications to manual therapy (e.g. connective tissue disorder, systemic inflammation). The photos for the vignettes were taken using the human volunteers, so each volunteer’s physical characteristics exactly matched the vignette (e.g. age, sex, height, weight, body shape). Each volunteer was screened (medical history and physical examination targeted to the thoracic spine) on each day of data collection by one of the authors (LG), a registered chiropractor with 12 years experience. The thoracic vertebra T6 was the target vertebra for all interventions and was marked by NT, a registered physiotherapist with 14 years experience.

### Equipment and outcome measures

**Fig. 1:**
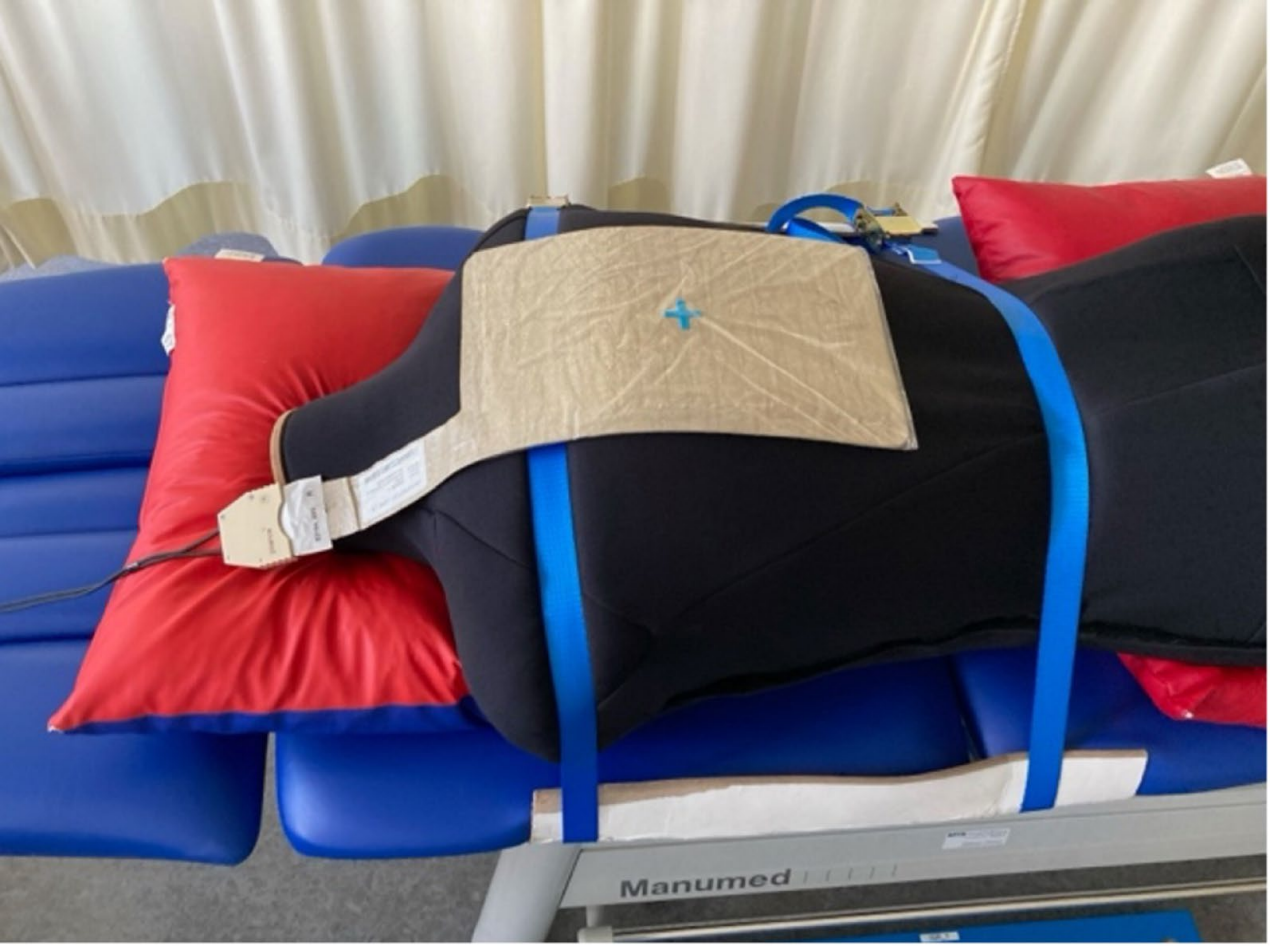
Manikin with pressure pad. The blue cross represents the marked vertebra T6

A human analogue manikin (HAM™; Canadian Memorial Chiropractic College, Toronto, Ontario) with an embedded spine [22] was securely strapped in a prone position to a height-adjustable treatment table. Perpendicularly applied forces at the clinician-recipient interface were measured using a thin flexible pressure pad (Novel pliance®, Munich, Germany; sampling rate 100 Hz (Hz)). The pad’s area was approximately 24 cm × 15 cm × 0.02 cm with a matrix of 10 × 16 sensors (size of each sensor: 1.5 cm × 1.5 cm) and the sensors were calibrated to a peak pressure of 1.2 megapascal (MPa) each. The sensor was positioned between the clinician’s hand and the recipient during MOB/MAN (Fig. 1). A sampling rate of 100 Hz has been shown to be adequate for capturing the force–time characteristics of MAN [32].

Primary outcomes were: (i) average peak force of MOB peaks during 30 seconds (s) (sum of all peaks in 30 s, divided by number of peaks) in Newton (N), and (ii) MAN total peak force (N) as the greatest force reached during the thrust, measured from a zero-force baseline.

Secondary outcomes were: (i) number of MOB peaks in 30 s, and (ii) MAN rate of force application (Newtons per second (N/s)), defined as peak force minus force at DIP (minimum force that appears after preload force and immediately before the onset of thrust) divided by time from DIP to peak, and (iii) time from DIP to peak for MAN.

### Study protocol

After written informed consent was given, demographic data of the included clinicians were captured using a REDCap® survey (Additional File 4). The definition of a Grade 3 MOB and MAN used in this study was explained to the clinicians (“For this study, we defined Grade 3 mobilization as a large amplitude movement which starts just before resistance 1 and ends at the end of physiological range of motion. We defined manipulation as a high velocity, low amplitude thrust technique where the articulation is moved past its physiological range of motion.”). Familiarization involved an introduction to the experimental setup and the delivery of both MOB for 15 s and a single MAN (order randomized). Treatment table height was adapted according to individual preference and remained constant for all following interventions. Both MOB and MAN, and the order of human and manikin, were determined according to a pre-generated randomization schedule. For MOB, a Grade 3 central posterior-to-anterior MOB was delivered for 30 s. For MAN, a single high velocity, low amplitude thrust was delivered.

Both MOB and MAN were delivered to both the humans and manikin for all three vignettes. Importantly, clinicians were instructed to use their preferred technique and hand positioning but to be consistent between both the human and manikin for all vignettes. They could, however, adjust the applied force or amplitude as they felt appropriate for the 'recipient'. After familiarization, data collection always started with vignette 2. This middle-aged scenario was chosen as a starting point to avoid beginning with the physiological extremes. The order of the subsequent two vignettes (1 and 3) was randomized and counterbalanced to avoid any learning or fatigue effects.

During data collection, no feedback was given regarding biomechanical characteristics.

Following data collection, each clinician answered an exit survey in REDCap**®** (Additional File 5). This survey gathered qualitative feedback on their experience. For example, clinicians were asked to compare the feel of delivering techniques on the manikin vs the human, to note any differences in their approach or comfort, and to report any difficulties encountered. A maximum of five clinicians were measured per day.

### Data analysis

All collected data were processed using Python 3.10 (Python Software Foundation TM). Statistical analysis was conducted in the Python library *scipy*.

For MOB, peak forces were identified in a three step procedure. First, leading and trailing zero-measurements were cut from the samples and peaks were identified as points with higher force readings than neighboring values. These candidate points had to also fulfill two further criteria: (i) a minimum temporal separation of 0.02 s between peaks (peaks closer than this were merged and the smaller one discarded); and (ii) a relative height filter (peaks had to exceed the 70% quantile of the force distribution after zeros were removed). In a second step, to account for the differences in MOB techniques between subjects, a relative distance measure (75% of the average distance between peaks) was used to clean twin peak candidates a second time. Troughs were then identified as minima between the cleaned peaks. Finally, through visual inspection of graphical output, the automated identification of each measurement was approved or, where necessary, flagged to be re-run with slightly adjusted minimal distance or minimal height criteria. Finally, the average of all peak forces within 30 s of MOB and the number of peaks were extracted. For MAN, total peak force, DIP, and rate of force application were extracted. Total peak force was identified as the maximum force and DIP was found by tracing back from peak force to the next local minimum, which had to fulfill an inertia criterium to clean bumps in the acceleration that were mistaken for the DIP (for 0.03 s before the DIP, the force could not drop further). In trials where a distinct DIP was not obvious (i.e., if the force increased smoothly without a clear drop), DIP was defined as the force at the moment the thrust was initiated (the last data point before the sharp rise in force). Again, visual inspection of graphical output was applied to confirm the identified peak and DIP.

Clinician demographics (e.g. years of experience) and biomechanical characteristics (e.g. total peak force) were reported descriptively. Additionally, an exploratory analysis was performed to compare force outputs between the two physiological extremes (vignette 1 vs vignette 3). This was done to examine whether clinicians modulated their forces differently for a young vs an older patient on the manikin compared to the human. Specifically, the difference in peak forces between vignette 1 and vignette 3 for each participant was calculated and those differences between the human and manikin conditions were compared.

A quantitative content analysis was conducted on the open-ended questions of the two surveys answered by the included clinicians before and after data collection. Following an inductive approach, frequently mentioned keywords were identified and manually categorized. The responses provided by the clinicians were systematically classified, and the frequency of each category was subsequently determined.

## Results

### Participants

Fifteen physiotherapists were included in the study, two were excluded (n = 1 withdrew consent before completing data collection; n = 1 did not follow the protocol and applied a different technique than instructed). Each of the 13 clinicians performed one 30 s MOB and one MAN for each vignette on each type of recipient (human and manikin), totaling six MOB and six MAN trials per clinician (78 of each overall). All clinicians have held a title in Orthopaedic Manual Therapy (OMT) for more than two years (Table 1).

**Table 1:** Demographic information of clinicians

	Median	IQR
Gender: 7 females, 6 males	–	–
Age (years)	40	36–45
Weight (kilograms)	68	58–72
Height (centimeters)	173	168–176
Experience as a physiotherapist (years)	14	12–21
Experience with MAN (years)	5	5–12
MAN per week	1	1–5

IQR: Interquartile ranges; MAN: Manipulation.

### More peak force applied to the manikin than the humans

**Fig. 2:**
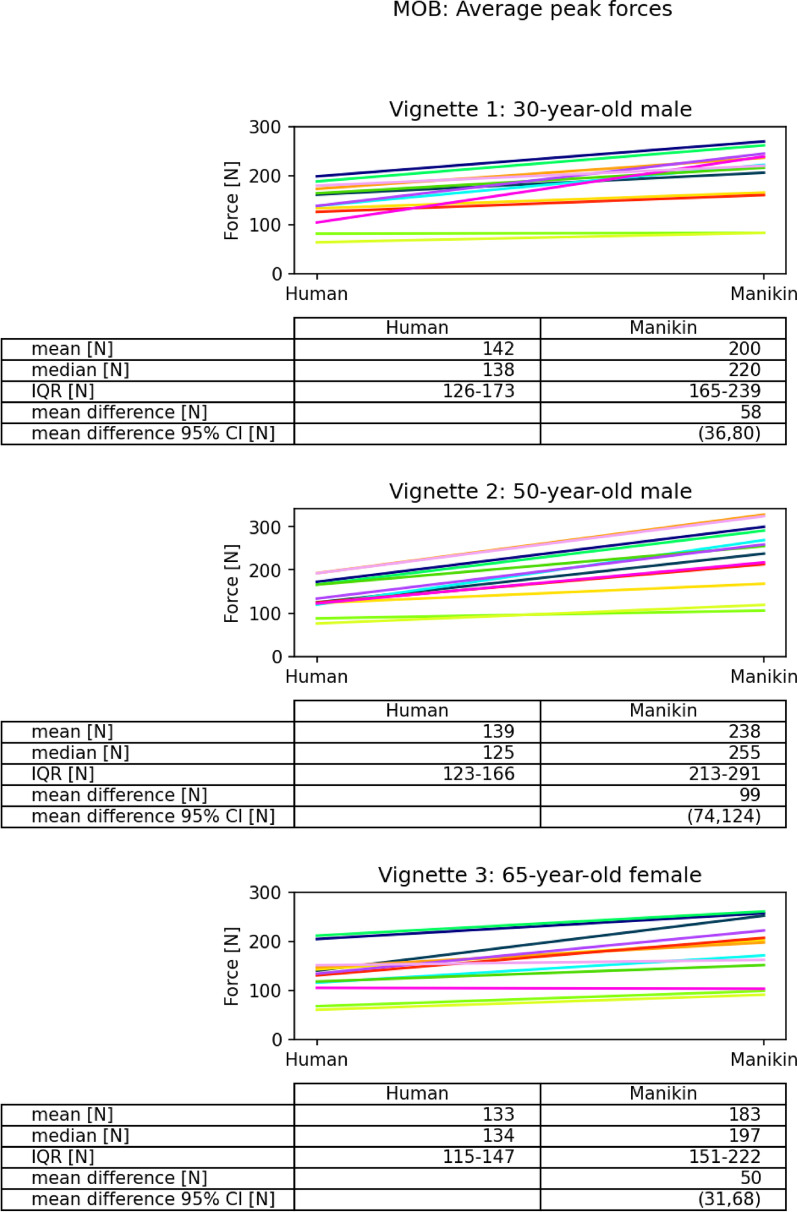
Grade 3 spinal mobilization. Colored lines represent data from each clinician. MOB: Mobilization; N: Newton; IQR: Interquartile Ranges; CI: Confidence Interval

**Fig. 3:**
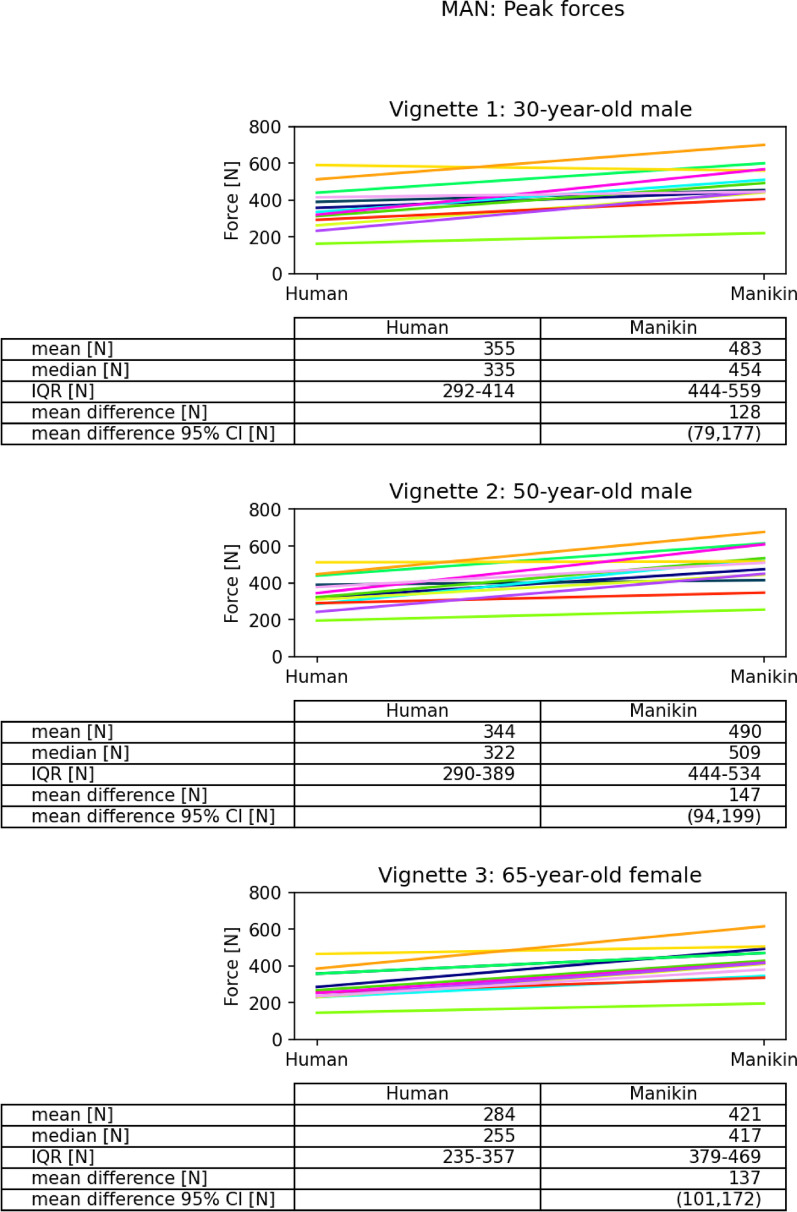
Spinal manipulation. Colored lines represent data from each clinician. MAN: manipulation; N: Newton; IQR: Interquartile Ranges; CI: Confidence Interval

Mean average peak force of 30 s MOB and mean total peak force of MAN on the manikin were higher than on the human for all vignettes (Figs. 2 and 3). The difference was largest for vignette 2 (50-year-old male) for MOB and MAN with a mean difference of 99N (95% confidence interval (CI) 74, 124) and 147N (94, 199), respectively. For vignette 1 (30-year-old male) the mean difference was 58N (36, 80) for MOB and 128N (79, 177) for MAN. For vignette 3 (65-year-old female) the mean difference was 50N (31, 68) and 137N (101, 172) for MOB and MAN, respectively.

### MAN total peak forces modulated according to the vignettes

Comparing the inter-vignette peak forces (for MAN) for the physiological extremes (vignette 1 versus vignette 3) showed that greater total peak forces were applied on the 30-year-old male than on the 60-year-old female. Interestingly, the mean difference in total peak force between vignette 1 versus 3 on the humans was similar to the manikin being 71 ± 51.8N on the humans and 62 ± 60.7N on the manikin (Table 2).

**Table 2:** Differences of peak forces between vignette 1 and 3

	Mean	Median	IQR	SD
MOB: average peak force differences [N]				
Human V1–V3	9	4	− 5 to 22	18.9
Manikin V1–V3	18	13	− 16 to 51	52.1
Difference in difference	8.4	18.5	− 27 to 24	49.3
MAN: peak force differences [N]				
Human V1–V3	71	67	33 to 105	51.8
Manikin V1–V3	62	65	30 to 85	60.7
Difference in difference	− 8.5	7.5	− 47 to 35	64.8

IQR: Interquartile Ranges; SD: Standard Deviation; N: Newton; V: Vignette; MOB: Mobilization; MAN: Manipulation.

In contrast, no real inter-vignette difference could be identified for average peak forces in MOB (Table 2). Mean difference in average peak force between vignette 1 and 3 on the human was 9 ± 18.9N and on the manikin 18 ± 52.1N. The ‘difference in difference’ was 8.4N.

### More peaks and higher rate of force application on the manikin

The number of peaks during MOB were higher on the manikin for all vignettes with a small difference of either 0.7 (vignette 1: ± 1.38) or 1.2 (vignette 2: ± 1.52; vignette 3: ± 2.48) peaks, respectively (Table 3). The lowest number of peaks was applied to vignette 3 with 15.2 ± 10.56 peaks on the human and 16.4 ± 9.43 peaks on the manikin.

**Table 3:** Secondary outcomes: average number of peaks and rate of force application

	Human	Manikin	Difference
MOB: mean average number of peaks (SD)			
Vignette 1	17.1 (11.32)	17.8 (11.57)	0.7 (1.38)
Vignette 2	16.5 (10.97)	17.7 (11.51)	1.2 (1.52)
Vignette 3	15.2 (10.56)	16.4 (9.43)	1.2 (2.48)
MAN: mean rate of force application [N/s] (SD)			
Vignette 1	1327 (849.0)	2479 (1051.9)	1152 (808.2)
Vignette 2	1450 (1020.7)	2582 (938.2)	1131 (1075.1)
Vignette 3	952 (670.1)	1961 (989.3)	1008 (867.9)
MAN: mean time from DIP to peak [ms] (SD)			
Vignette 1	180 (70.8)	150 (45.5)	-30 (89.4)
Vignette 2	250 (199.8)	140 (43.5)	-100 (181.2)
Vignette 3	230 (115.9)	180 (85.3)	-50 (67.8)

SD: Standard Deviation; N/s: Newtons per second; MOB: Mobilization; MAN: Manipulation; ms: milliseconds.

Rate of force application (N/s) during MAN was higher on the manikin for all vignettes (Table 3). On the humans, thrusts were delivered with the highest rate of force application to vignette 2 (1450 ± 1020.7N/s) and the lowest to vignette 3 (952 ± 670.1N/s). Similarly, on the manikin, the highest rate of force application was measured on vignette 2 (2582 ± 938.2N/s) and the lowest on vignette 3 (1961 ± 989.3N/s).

Mean time from DIP to peak for MAN was longer on the human than on the manikin for all vignettes (Table 3). The largest difference was on vignette 2 (100 ± 181.2 ms (ms)), with a mean DIP-to-peak time of 250 ± 199.8 ms on the human, representing the longest mean time observed across all procedures.

### Feedback from the clinicians

In the post-data collection questionnaire, 12 of the 13 (92.3%) clinicians reported that the delivery of both MOB and MAN to the manikin was different to those applied to the humans (Table 4). The remaining clinician, who indicated no difference, still noted in their comments that the manikin was noticeably stiffer than the humans (Additional File 6).

**Table 4:** Post-data collection questionnaire

	Yes	No
Do you think you applied mobilization and manipulation similarly between the human volunteers and the manikin?	1	12
Was your treatment comparable to the treatments you do in daily practice?	4	9
Did you intend to modulate the forces according to the 'Patient' you treated?	11	2

Additionally, nine clinicians (69.2%) felt that the MOB and MAN delivered for this study differed from that which they deliver in daily clinical practice because it was not possible to communicate with the humans/manikin, there was no direct skin contact, palpation and joint play could not be assessed, and no feedback was received from the humans/manikin.

The majority of the clinicians (n = 11, 84.6%) reported attempting to modulate the forces according to the vignettes presented to them.

### Adverse events

Transient mid-thoracic spine pain was reported by one human volunteer after the final MOB intervention of that day of data-collection. According Carnes et al. [31] it was classified as a ‘mild and not adverse’ adverse event as the discomfort was mild and resolved within 24 h without requiring treatment. No other adverse effects were observed in any participant.

## Discussion

This is the first study to compare the force–time characteristics of MOB and MAN delivered to the thoracic spine of a manikin to those delivered to a human by experienced clinicians. Average peak force and number of peaks during MOB and total peak force and rate of force application of MAN were higher on the manikin than on the humans. Similarly, Duquette and colleagues [28] reported total peak forces of cervical spinal MAN, delivered by students, to be approximately twice as large on the manikin than on the human. However, forces were measured at the recipient-table interface and not directly at the clinician-recipient interface (as in the present study) and it is unknown how forces transmit through the recipient to the table. Indeed, higher forces have previously been measured at the recipient-table vs. clinician-recipient interface [33]. Furthermore, it is likely that force transmission differs between humans and manikins. As such, the results of the two studies are not directly comparable.

There are several possible reasons why clinicians applied more force to the manikin vs. humans. In the post-data collection questionnaire, 12 of 13 clinicians (92.3%) indicated that the delivery of MOB and MAN to the manikin differed from humans, while the remaining clinician also remarked on its increased stiffness. Notably, seven clinicians (53.9%) specifically reported that the manikin was stiffer and required greater force to perform MOB and MAN. This may explain the higher forces recorded on the manikin; rather than replicating the forces they applied to humans, clinicians instead responded to the lack of tactile feedback provided by the manikin. The manikin’s mechanical properties therefore elicited greater force output. This suggests that the results reflect a combination of conscious adaptation to the vignettes and unconscious response to the manikin’s stiffness, highlighting its limited tactile fidelity. This point is salient as it is possible that a manikin with a more flexible spine (i.e. with human-like force–displacement properties) could result in the application of forces more similar to those delivered to the humans. This hypothesis is supported by the work of Descarreaux and colleagues [34] who instrumented a cardiopulmonary reanimation manikin with a spring to imitate the resistance of a thoracic spine of a prone-positioned patient. It was reported that mean peak force and rate of force application were similar to the values measured in other studies [35–37] when delivering thoracic MAN to humans. More recently, Owens and colleagues [38] developed a manikin and tested the thoracic spine posterior-to-anterior stiffness. The stiffness was reported to be in the range of that found in humans, suggesting that this manikin might have greater tactile fidelity than the manikin used in the current study. Future research should compare the biomechanical characteristics of MOB and MAN delivered to both humans and manikins with different tactile fidelities to investigate similarities and differences in applied forces.

Secondly, it is possible there could have been a lack of consideration for patient comfort when MOB and MAN were delivered to the manikin. The question of modulating force application during MAN to address recipient comfort has been discussed in terms of both manikins [28] and cadavers [39]. Duquette and colleagues [28] hypothesize that the total peak forces applied on humans are lower because of the clinicians’ reluctance to thrust on a live human. Additionally, Symons and colleagues [39] reported that the effect of basal muscle tone in living humans may change force transmission through the body. However, no research on this topic exists for MAN or MOB delivered to any spinal region.

This is the first study comparing MOB and MAN delivered by physiotherapists to humans and a manikin. Therefore, we focused on experienced clinicians with post-graduate training in MAN to ensure they had adequate psychomotor skill competency to deliver both MOB and MAN. The peak forces for MOB observed in this study were lower than those reported in existing literature. Gorrell et al. [14] summarized findings from two studies involving 50 participants, which found peak forces between 297 and 323 N [40, 41]. Yet, comparison is limited as the mobilization grade was not specified, and in one of the two studies the interventions were delivered by chiropractors to older adults, while the other study did not report the practitioners’ professional background. In contrast, the total peak forces for MAN fall within the wide range described in the literature where total peak forces of thoracic MAN on humans at the clinician-patient interface has been reported to span from 212 to 1213 N [18]. Similarly, a recent study by Nyirö et al. [42] found total peak forces of thoracic MAN delivered by chiropractors to a manikin ranging between 89 and 1285 N.

Although the dosage of biomechanical characteristics of MOB and MAN required for clinical relevance is not clear [13–16, 18, 20, 22, 23], research shows that experienced clinicians apply more force, more quickly, than students during both MOB and MAN [34, 43, 44]. Furthermore, in the teaching of chiropractic students, manikins are increasingly used for the acquisition and development of MAN skills [22, 24]. There are several advantages for manikin use in the classroom, including: MAN can be trained repeatedly without exposing the recipient to repeated end-range thrusting, and MAN can be quantified and real time feedback can be provided to students when manikins are used in combination with force-sensing technologies. Despite these advantages, there exists no published literature comparing similarities and differences between MOB delivered to humans and manikins, and only one study from Duquette and colleagues making the comparison for MAN [28]. Futhermore, no literature exists investigating if manikins could be used to scaffold students from MAN skills acquisition and development to force modulation based on imagined clinical vignettes. In our study, total peak forces of MAN applied to the patient scenario of the young man were higher than to the elderly woman, no matter if the techniques were delivered to the human or to the manikin. Additionally, not only total peak force of MAN was lowest on vignette 3, but also average number of peaks of MOB and rate of force application of MAN. This suggests that, overall, the experienced clinicians included in this study were able to modulate their applied forces based on an imagined clinical vignette, even when delivering MOB and MAN to a manikin. However, there was inter-clinician variability in this modulation, with some clinicians applying nearly the same forces across vignettes, whereas others adjusted their force substantially between the young and elderly patient scenarios (Figs. 2 and 3). Supporting this finding, two recent studies have reported that clinicians modulate force application based on provided clinical vignettes [42, 45] when delivering MAN to a manikin/mechanical aids. Therefore, when considering the use of manikins for the scaffolding of students from MAN and/or MOB skills acquisition and development to force modulation based on imagined clinical vignettes, it is essential to be aware that the applied forces might be different on a manikin than on humans. To our best knowledge, manikins are not used in the teaching of manual technique skills to physiotherapy students. This highlights a possible new role for manikins, especially in combination with real time feedback using force-sensing technology, in physiotherapy curricula [22]. Future research in this area is necessary, involving both physiotherapy and chiropractic students to evaluate how MAN and MOB skills gained from training on a manikin transfer to the delivery of these interventions to live humans.

### Strengths and limitations

This study analyzed data of a small number of experienced physiotherapists. All reasonable attempts were made to recruit more participants, with over 150 physiotherapists directly contacted and information widely distributed via social media, flyers, and word-of-mouth advertising. Additionally, all swiss physiotherapists with an OMT title were invited to participate in this study via a newsletter sent by their professional association. However, as this was an exploratory study, we believe that the results are still relevant. Including experienced clinicians with the necessary post-graduate training decreased the likelihood of inappropriate application of MAN and MOB. Additionally, including MAN allowed to put our findings in context with other research utilizing manikins as there is sparse literature on this topic for MOB.

Despite clear and standardized instructions being given and sufficient time for familiarization being provided, the experimental equipment (e.g. pressure pad) used to quantify MOB and MAN could have influenced force application during the interventions. The pressure pad was placed between the clinician’s hand and the spine of the recipient. This likely changed the tactile feedback. Still, the use of the pressure pad has an essential advantage because the applied forces are measured directly at the clinician-recipient interface. Because each clinician contributed only a single trial per scenario, the results do not capture within-clinician variability. With repeated trials, some clinicians might demonstrate greater consistency or converge toward a characteristic force value. Still, in actual clinical practice, a single thrust is typically delivered when treating a patient. Furthermore, it has been shown that repeatability of a MAN on the thoracic spine of a manikin delivered by chiropractors was fair to excellent, with force–time characteristics remaining very consistent [42]. Moreover, ensuring the comfort of the human volunteers in this study required limiting the number and intensity of thrusts to only what was necessary. Human volunteers and the manikin could only be treated in a prone position and 3 (23.1%) of the clinicians reported that in clinical practice they would deliver MAN in a supine position and 4 (30.8%) would usually lean on the treatment table, which was not allowed in this study because clinician ground reaction forces during MOB and MAN were measured using two force plates on which the clinicians stood during data collection. However, these data are not reported in this manuscript. For 12 of the 13 clinicians (92.3%) measured, it was the first time they delivered manual techniques to a manikin.

## Conclusion

In this study, force–time characteristics of MOB and MAN performed by experienced physiotherapists on the thoracic spine of a manikin were different from those delivered to healthy humans: the forces applied to the manikin were higher for all vignettes for both interventions. This exploratory study provides unique data on this topic and suggests that the experienced clinicians participating in this study were able to modulate total peak force of MAN delivery to an imaginary clinical scenario, regardless of whether their delivery was to a manikin or human. Knowing the advantages of using a manikin to train the complex psychomotor skills of manual techniques, future research should assess the lack of evidence surrounding the transfer of skills learned by students delivering manual therapy interventions on a manikin in the classroom to clinical practice. If it can be shown that manikin-based training of manual techniques is relevant for the use in clinical practice on patients, this teaching aid should be considered for physiotherapy education.

## Supplementary Information

Below is the link to the electronic supplementary material.

Supplementary Material 1

Supplementary Material 2

Supplementary Material 3

Supplementary Material 4

Supplementary Material 5

Supplementary Material 6

Supplementary Material 7

## Data Availability

The datasets used and/or analysed during the current study are available from the corresponding author on reasonable request.

## Abbreviations

DIP: Downward incisural point
CI: Confidence interval
Hz: Hertz
IQR: Interquartile ranges
MAN: Manipulation
MOB: Mobilization
MPa: Megapascal
ms: Milliseconds
N: Newton
Nr: Number
N/s: Newtons per second
Obs: Observations
OMT: Orthopaedic manual therapy
s: seconds
SD: Standard deviation
V: Vignette
