# Supplementary material for: Force variability of thoracic spine mobilization and manipulation delivered by experienced physiotherapists to healthy human volunteers and a manikin: an observational study
Source: Chiropr Man Therap. 2025 Dec 9;33:56. doi: 10.1186/s12998-025-00619-7 (PMC12690789; doi:10.1186/s12998-025-00619-7)
Supplement: Supplementary file 3 — Supplementary Material 3 [file 12998_2025_619_MOESM3_ESM.pdf]

Imagine the following scenario:

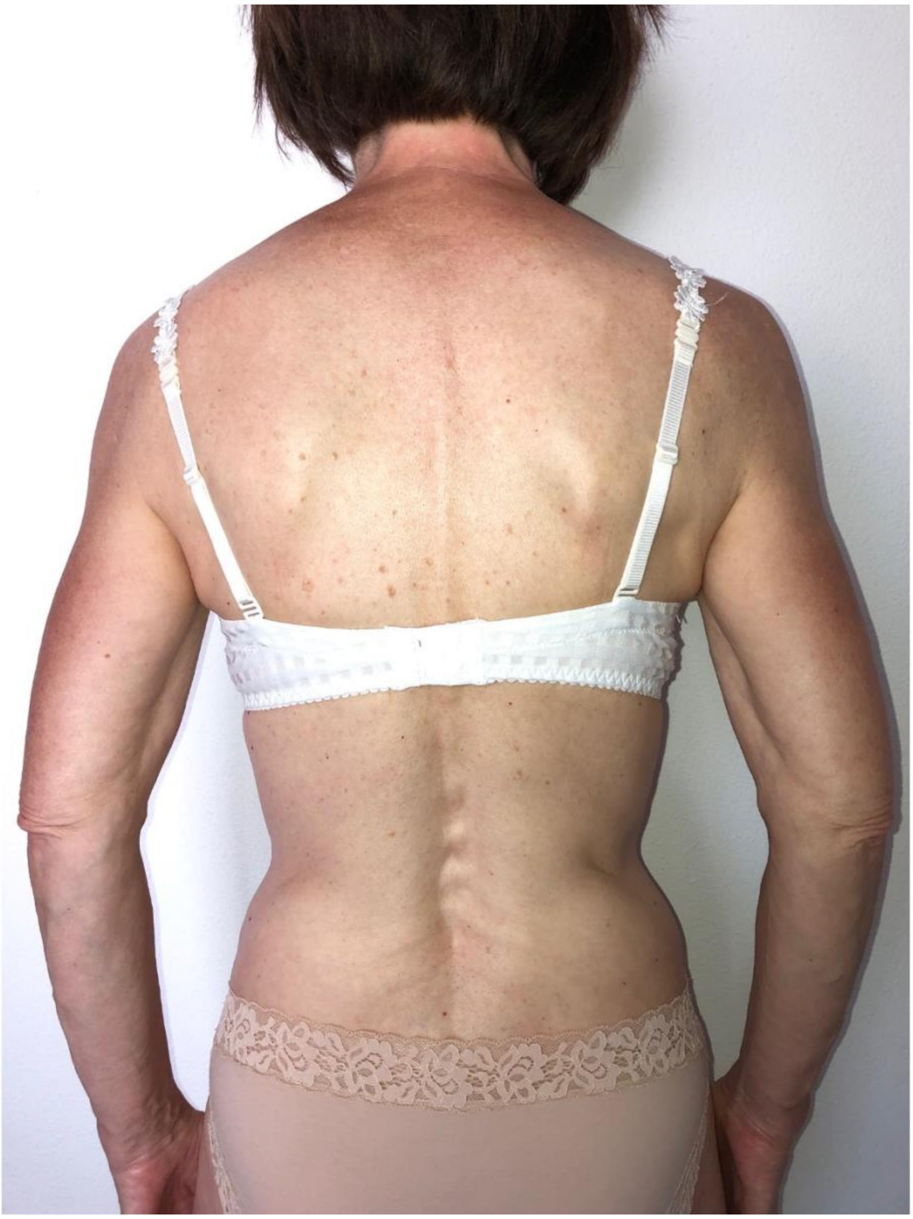

- Healthy female patient
- Age: 65 years old
- Height: 165cm
- Normal body weight for height
- Responded well to previous manual therapy on mid-thoracic spine
- No radiation, no signs or symptoms of inflammatory or degenerative pathologies
