# Supplementary material for: Force variability of thoracic spine mobilization and manipulation delivered by experienced physiotherapists to healthy human volunteers and a manikin: an observational study
Source: Chiropr Man Therap. 2025 Dec 9;33:56. doi: 10.1186/s12998-025-00619-7 (PMC12690789; doi:10.1186/s12998-025-00619-7)
Supplement: Supplementary file 4 — Supplementary Material 4 [file 12998_2025_619_MOESM4_ESM.pdf]

Pre-data collection questionnaire

Demographics

Gender

Year of birth

Handedness

Height in cm (please measure)

Weight in kg (please measure)

Work experience

Years since graduation as a physiotherapist

Have you been a significant (3 months or more) time off as a physiotherapist? (e.g. because of pregnancy, motherhood, illness, travelling etc)

What was the reason for your absence?

How many months?

Since how many years have you been manipulating on the spine?

What kind of further training in manipulation did you complete (e.g. Maitland, SAMT, OMT)?

Do you have an OMT-title?

Since how many years do you have an OMT-title?

How many times (on average) do you deliver spinal manipulation per week?

Have you been a significant (3 months or more) time off as an OMT? (e.g. because of pregnancy, motherhood, illness, travelling etc)

What was the reason for your absence?

How many months?

Percentage employed as a physio:

Please provide the definition you use for a grade 3 spinal mobilization:

Please provide the definition you use for a spinal manipulation:

Do you have any experience delivering spinal mobilization and/or manipulation to a manikin?
