# Supplementary material for: Force variability of thoracic spine mobilization and manipulation delivered by experienced physiotherapists to healthy human volunteers and a manikin: an observational study
Source: Chiropr Man Therap. 2025 Dec 9;33:56. doi: 10.1186/s12998-025-00619-7 (PMC12690789; doi:10.1186/s12998-025-00619-7)
Supplement: Supplementary file 5 — Supplementary Material 5 [file 12998_2025_619_MOESM5_ESM.pdf]

Post-data collection questionnaire

Do you think you applied mobilization and manipulation similarly between the human volunteers and the manikin?

Why was it similar?

What was different?

Was your treatment comparable to the treatments you do in daily practice?

Why was it similar?

Why not?

Did you do a grade 3 mobilization as it was defined in this study? (Large amplitude movement which starts just before resistance 1 and ends at the end of physiological range of motion.)

What is the reason?

Did you do a manipulation as it was defined in this no study? (High velocity, low amplitude thrust technique where the articulation is moved past its physiological range of motion.)

What is the reason?

Did you intend to modulate the forces according to the 'Patient' you treated?

Any further comments regarding data collection or anything else you would like to share with us?
