# Supplementary material for: Force variability of thoracic spine mobilization and manipulation delivered by experienced physiotherapists to healthy human volunteers and a manikin: an observational study
Source: Chiropr Man Therap. 2025 Dec 9;33:56. doi: 10.1186/s12998-025-00619-7 (PMC12690789; doi:10.1186/s12998-025-00619-7)
Supplement: Supplementary file 6 — Supplementary Material 6 [file 12998_2025_619_MOESM6_ESM.pdf]

Post-data collection questionnaire

Do you think you applied mobilization and manipulation similarly between the human volunteers and the manikin?	Why was it similar?	What was different?	Was your treatment comparable to the treatments you do in daily practice?	Why was it similar?	Why not?	Did you do a grade 3 mobilization as it was defined in this study? (Large amplitude movement which starts just before resistance 1 and ends at the end of physiological range of motion.)	If no- What is the reason?	Did you do a manipulation as it was defined in this no study? (High velocity, low amplitude thrust technique where the articulation is moved past its physiological range of motion.)	If no- What is the reason?	Did you intend to modulate the forces according to the 'Patient' you treated?	Further comments
no		The resistance of the manikin was noticeably higher than that of the patients. In addition, no respiratory movements could be felt in the manikin. The anatomy also differed, making hand placement, particularly for manipulation, more difficult.	no		This applies particularly to manipulation. In clinical practice, I provide patients with significantly more instructions (e.g., regarding pressure and inhalation/ exhalation), which in my view influences the quality of the manipulation.	yes		Yes		no	Regarding the last question: I tried to adapt the treatment to the resistance I felt in the individual patients. The written information (e.g., height, weight) was of secondary importance, particularly since all patients were healthy. I would have relied more on the written information if, for example, specific comorbidities had been present.
No		he feedback under my hands during pressure build-up	Yes	because the application primarily relies on the feedback from my hands		Yes		Yes		Yes	

no		Resistance of the spine and amplitude up to the end-feel	No		I usually perform thoracic spine manipulation with the patient in the supine position.	yes		Yes		Yes	
yes	My force application was the same, but the manikin was considerably stiffer.		No		I wouldn't perform mobilization for that long	no	Perhaps I moved further back, almost completely losing contact with the resistance.	Yes		yes	The manikin was very stiff. I treated both men with the same force, while I tried to be less forceful with the woman..
No		The resistance of the manikin was noticeably higher than that of humans.	yes	The hand placement and body position were the same.		Yes		yes		yes	In clinical practice, I would have braced my legs on the edge of the treatment table to position myself more centrally over the patient. Additionally, the hand contact with the pad was not as direct or intensive as it would be on the patient's skin.
No		Interventions were easier on the manikin	No		Use the mobilization wedge instead of my fingers.	Yes		no	Manipulation was easier on the manikin. In clinical practice, I use the mobilization wedge.	yes	
no		On the manikin, R1 and R2 are much closer together.	No		Normally, I brace my thighs against the treatment table.	Yes		Yes		yes	The pad is slippery, making it impossible to apply skin tension
No		The curvature of the kyphosis and the relief of the back muscles—on the manikin, there was noticeably better	No		No joint play test was performed beforehand, no communication with the	Yes		yes		no	I adjust the intensity based on the joint play and what the patient can tolerate.

		support, and the height of the mobilization site felt different.			patients regarding pressure intensity, and the table could not be braced with the legs.						
No		The resistance felt different, and I probably applied more pressure.	No		Starting position with the knee on the table.	Yes		no	In Vignette 1, I did not go beyond R2—it was too slow and somewhat weak.	yes	
no		The pad is confusing and offers less orientation, with a tendency to slip under the hands; on the manikin, the end-feel is not the same, landmarks (joint, height) could not be palpated, and breathing could not be utilized.	no		In clinical practice, one knows the patients, can palpate beforehand, is familiar with the resistance/end-feel of the segment to be treated, observes the patient's responses, can compare with the segments above and below, and has a specific treatment goal.	yes		yes		yes	It would be helpful to know how many repetitions are planned and to possibly perform the palpation oneself to anticipate what to expect. Placing the hands on the pad without slipping is difficult, and maintaining a stable stance on the force plates can be challenging.
No		The second resistance on the manikin felt different and was much shorter. However, I tried to apply the same amount of pressure.	yes	I use the techniques in the same way in clinical practice.		Yes		Yes		yes	
No		The resistance of the manikin was significantly higher	no		Since I did not have direct skin contact.	Yes		yes		yes	

		than that of humans.									
no		The resistance was very different. The examiner said during the last patient: “Keep the previous patient in mind during mobilization on the manikin.” Then it felt similar. But normally, we adjust the treatment according to the resistance. Perhaps it would have been better to start with a human patient and then the manikin.	yes	Appropriate examples, common regions		yes		yes		yes	For the questions about whether it was the same or different, both answers could always apply. The difficulty was that, as far as I could feel, the manikin did not give at all, making it impossible to adjust the technique. If the patients had been treated first and we had been explicitly instructed, “Please perform the same technique on the manikin with the same intensity and amplitude,” a comparison would have been possible. Another challenge was that the setup only allowed treatments from the side of the table, so switching positions wasn’t possible. I also noticed that I often lean briefly on the table, which was not allowed here. Thank you for your work.
