# Supplementary material for: Force variability of thoracic spine mobilization and manipulation delivered by experienced physiotherapists to healthy human volunteers and a manikin: an observational study
Source: Chiropr Man Therap. 2025 Dec 9;33:56. doi: 10.1186/s12998-025-00619-7 (PMC12690789; doi:10.1186/s12998-025-00619-7)
Supplement: Supplementary file 7 — Supplementary Material 7 [file 12998_2025_619_MOESM7_ESM.pdf]

Kanton Zürich
Kantonale Ethikkommission

Prof. em. Dr. med. David Nadal
Präsident Abteilung A

Annette Magnin, M. Sc. ETH
Geschäftsführerin
Stampfenbachstrasse 121
Postfach
8090 Zürich
Telefon +41 43 259 79 70
Fax +41 43 259 79 72
admin.kek@kek.zh.ch
www.kek.zh.ch

Einschreiben
Universitätsklinik Balgrist
Integrative Spinal Research Group
Balgrist Campus
Dr. Lindsay Gorrell
Lengghalde 5
8008 Zürich

18. Juli 2023 / plm

Verfügung der Kantonalen Ethikkommission Zürich

**BASEC-Nr.**	2023-01094
**Projekttitel**	Mobilization and manipulation of the thoracic spine: Quantifying the variability of forces delivered to healthy human volunteers and manikins.
**Gesuchsteller/in**	Dr. Lindsay Gorrell, Integrative Spinal Research Group, Balgrist Campus, Zürich
**Zentren**	Dr. Lindsay Gorrell, Integrative Spinal Research Group, Balgrist Campus, Zürich

Entscheid

Auf das Gesuch wird nicht eingetreten.

Begründung: Gemäss E-Mailkorrespondenz vom 14.07.2023.

Bedeutet: Die Ethikkommission ist für die Beurteilung rechtlich nicht zuständig

Kontaktperson: Dr. sc. nat. Tobias Rosenberger

Entscheidverfahren

☐ ordentliches Verfahren ☐ vereinfachtes Verfahren ☒ Präsidialentscheid

Am Entscheid beteiligte Kommissionsmitglieder siehe Anhang.

Die Ethikkommission bestätigt, dass sie nach ICH-GCP arbeitet.

Gebühren

Betrag: CHF 200.- Tarifcode: 6.0

Gemäss der geltenden Gebührenordnung von swissethics.

Rechtsmittelbelehrung

Gegen diesen Beschluss kann innert 30 Tagen, von der Mitteilung an gerechnet, beim Regierungsrat des Kantons Zürich schriftlich Rekurs eingereicht werden. Die Rekursschrift muss einen Antrag und dessen Begründung enthalten. Der angefochtene Entscheid ist beizulegen oder genau zu bezeichnen. Die angerufenen Beweismittel sind genau zu bezeichnen und soweit möglich beizulegen.

Kopie an

- ☒ Sponsor
- ☐ Swissmedic
- ☐ Bundesamt für Gesundheit
- ☐ beteiligte Ethikkommission
- ☐ andere:

Prof. em. Dr. med. David Nadal
Präsident

Annette Magnin, M. Sc. ETH
Geschäftsführerin

Anhang: - Allgemeine Hinweise
- Eingereichte Dokumente
- Am Entscheid beteiligte Kommissionmitglieder der Abteilung A

Allgemeine Hinweise:

Weitere Bewilligungspflichten: Unterliegt das Forschungsvorhaben einer weiteren Bewilligungspflicht (Swissmedic/Bundesamt für Gesundheit), darf mit dessen Durchführung erst begonnen werden, wenn beide Bewilligungen vorliegen und diese auf identische Versionen der Gesuchsunterlagen beruhen. Stimmen die Versionen nicht überein, muss zur Angleichung der Dokumente eine Änderung eingereicht werden.

Einreichung Dokumente: Revidierte und neue Dokumente zum Forschungsvorhaben sollen ausschliesslich über das Web-Portal BASEC auf der entsprechenden Formularseite des betreffenden Gesuches eingereicht werden. Obsolete Dokumente sind zu entfernen und bei neuen oder angepassten Dokumenten sind Datums- und Versionsangaben entsprechend zu ergänzen. Die erfolgten Änderungen müssen im Korrekturmodus abgefasst und zusätzlich als Version ohne Markierungen eingereicht werden. Änderungen, die nicht markiert sind, werden nicht überprüft und sind nicht Gegenstand der Bewilligung. Die Studieninformationen und -einwilligungen, das Protokoll und die Änderungen müssen in MS Word- oder durchsuchbaren PDF-Dateien eingereicht werden, insbesondere müssen gescannte Dokumente eine Texterkennung durchlaufen haben (OCR). Die handschriftlich unterzeichneten Unterschriftenseiten sind in eingescannter Form einzureichen. Das unterschriebene und datierte Begleitschreiben muss die Antworten auf eventuell von der EK gestellte Fragen enthalten.

Sprachversionen: Die zuständige Ethikkommission überprüft im Rahmen des Bewilligungsverfahrens Unterlagen zur Rekrutierung, zur Aufklärung, zur Einwilligung und zur Erhebung von Daten in ihrer Amtssprache. Unterlagen in einer anderen Sprache werden von der Ethikkommission lediglich zur Kenntnis genommen. Für die korrekte Übersetzung ist der Sponsor oder die Projektleitung verantwortlich.

Registrierung klinischer Versuche: Der Sponsor ist verpflichtet, den klinischen Versuch in einem WHO-Primärregister oder im Register der Nationalen Medizinbibliothek der USA (clinicaltrials.gov) zu erfassen und anschliessend die Referenznummer des Registerbeitrags im BASEC-Portal unter dem Screen SNCTP einzugeben. Die Übertragung der erforderlichen Daten in das Swiss National Clinical Trials Portal (SNCTP) erfolgt nach Bewilligung der Ethikkommission und Zustimmung des Gesuchstellers automatisch. Die Informationen über den klinischen Versuch sind in beiden Registern öffentlich zugänglich.

Swissethics veröffentlicht zudem wenige Informationen wie Titel, Projekttyp oder Leit-Ethikkommission der durch die kantonalen Ethikkommissionen bewilligten Gesuche auf swissethics.ch (ausser klinische Phase-I-Arzneimittelversuche).

Eingereichte Dokumente für das Hauptzentrum

Dr. Lindsay Gorrell, Balgrist University Hospital, Zürich

Dokument	Dok.Datum	Version
**1. Cover Letter**		
cover-letter-nathalie-english-final.docx	12/06/2023	
**3. Participant information sheet and informed consent (ICF)**		
combined-pifs-in-both-languages.pdf	12/06/2023	1
**4. Study plan (protocol), signed and dated**		
basec-protocol-nathalie-final-signed.pdf	12/06/2023	1
**5. CRF (Case Report Form)**		
crf-nathalie-final.docx	12/06/2023	1
**6. Investigator's CV, dated**		
cv-gorrell.pdf	12/06/2023	
**8. Details on infrastructure suitability and availability at the location where the trial is executed**		
qualifikationpruefort-nathalie-german-final.docx	12/06/2023	
**10. Insurance**		
see doc/cat: **4**, page/ref: **15**		
**11. Other documents handed over to study participants**		
flyers-recruitment-scripts-clinician-questionnaires-final-for-basec-submission.pdf	12/06/2023	1
**12. Details on nature and scope/value of compensation for participants**		
The Patient Information Form (Document no. 3) contains details on compensation		
**14. Information on secure handling of biological material and personal data, and in particular on the storage thereof**		
see doc/cat: **4**, page/ref: **16**		

Zusammensetzung der am Entschied beteiligten Kommission

Name, Vorname	Am Entscheid beteiligt
Prof. em. Dr. med. Nadal, David	☒
Dr. theol. Baumann-Hölzle, Ruth	☐
PD Dr. med. Betschart, Cornelia	☐
Dr. med. Bridler, René M.H.A.	☐
PhD MNSc Geschwindner, Heike	☐
David Haerry	☐
PD Dr. med. Andreas Hötker	☐
PD Dr.med. Dr.med.dent. Jacobsen, Christine	☐
Dr. Jeker, Raphael	☐
Prof. Dr. med. Jetter, Alexander	☐
Prof. Dr. rer. nat. Dipl.-Psych. Jokeit, Hennric	☐
Kapossy, Katrin, Fürsprecherin	☐
Dr. Keller-Senn, Anita PhD	☐
PD Dr. med. Künig, Gabriella	☐
Prof. em. Dr. med. Felix Niggli	☐
Dr. iur. RA Mausbach, Julian	☐
Dr. med. Muff, Brigitte	☐
Prof. Dr. med. Pestalozzi, Bernhard	☐
Prof. Rauch, Anita	☐
Prof. Dr. phil. Siegrist, Michael	☐
Prof. Dr. med. Spinas, Giatgen	☐
Prof. Dr. med. Stocker, Reto	☐
Lic. phil. Ziltener, Erika	☐
